# Predicting disease genes based on multi-head attention fusion

**DOI:** 10.1186/s12859-023-05285-1

**Published:** 2023-04-21

**Authors:** Linlin Zhang, Dianrong Lu, Xuehua Bi, Kai Zhao, Guanglei Yu, Na Quan

**Affiliations:** 1 College of Software Engineering, Xinjiang University, Urumqi, China; 2 College of information Science and Engineering, Xinjiang University, Urumqi, China; 3 Medical Engineering and Technology College, Xinjiang Medical University, Urumqi, China

**Keywords:** Pathogenic gene prediction, Heterogeneous network, Multi-head attention, Graph representation learning

## Abstract

**Background:**

The identification of disease-related genes is of great significance for the diagnosis and treatment of human disease. Most studies have focused on developing efficient and accurate computational methods to predict disease-causing genes. Due to the sparsity and complexity of biomedical data, it is still a challenge to develop an effective multi-feature fusion model to identify disease genes.

**Results:**

This paper proposes an approach to predict the pathogenic gene based on multi-head attention fusion (MHAGP). Firstly, the heterogeneous biological information networks of disease genes are constructed by integrating multiple biomedical knowledge databases. Secondly, two graph representation learning algorithms are used to capture the feature vectors of gene-disease pairs from the network, and the features are fused by introducing multi-head attention. Finally, multi-layer perceptron model is used to predict the gene-disease association.

**Conclusions:**

The MHAGP model outperforms all of other methods in comparative experiments. Case studies also show that MHAGP is able to predict genes potentially associated with diseases. In the future, more biological entity association data, such as gene-drug, disease phenotype-gene ontology and so on, can be added to expand the information in heterogeneous biological networks and achieve more accurate predictions. In addition, MHAGP with strong expansibility can be used for potential tasks such as gene-drug association and drug-disease association prediction.

## Background

Gene mutation and abnormal expression are usually the key factors that cause disease. Predicting disease genes is greatly significant for the diagnosis of human disease. With the rapid development of DNA sequencing technology, more and more biological databases are established, which provide sufficient data for the study of pathogenic genes. Many studies have confirmed that there is a complex cross-regulation relationship among diseases, genes, lncRNAs, and miRNAs. MiRNAs and lncRNAs play an important role in developing complex human diseases [1, 2]. Using multi-omics data and computer technology to predict pathogenic genes has become a research hotspot in recent years.

So far, traditional approaches, using gene expression, genome-wide association studies (GWAS) or clinical trials, are useful for discovering disease-related genes [3–6]. However, these methods are time-consuming and costly. Methods, using gene similarity, have been proposed successively to overcome this issue. For example, the Katz measure method [7], the gene-specific score method [8], the shortest path method [9] and the Endeavour method rely on the guilt-by-association concept [10]. These methods work under the hypothesis that genes with similar functions are more likely to be related to similar diseases. Therefore, it is necessary to develop computational methods which do not depend on the known gene-disease association information to identify disease-causing genes. Recently, Machine Learning (ML) has been widely used in predicting disease genes. Matrix factorization (MF) is a strategy to fill partially observed matrix. The methods based on MF have been used to discover unknown disease-related genes and achieved better performance [11–13]. These MF algorithms usually require a lot of computing power. Most algorithms can only handle limited data types, and the prediction performance is affected by the amount of data. The kernel function is a method to transform nonlinear data in original data space into high-dimensional linearly separable data, which has made great achievements in gene-disease association prediction [14–16]. Nonetheless, these kernel methods only focus on the single trait of genes but ignore biodiversity, and are incomplete in extracting gene features. The methods of combining Laplace with random walk [17–20] have achieved success in the prediction of pathogenic genes. In addition, He and Li et al. [21, 22] compared and analyzed the performance results of different machine learning methods used for predicting disease genes. However, with the rapid growth of biological data in recent years, the above methods still have challenges in effectively dealing with the sparsity of biological networks and still have certain constraints in specific applications.

As a kind of advanced technology in the field of machine learning, deep learning methods can quickly and efficiently process unstructured data and efficiently extract potential features from complex networks. For example, graph convolutional neural network methods using multi-source data extract features from heterogeneous networks to predict disease-causing genes [23–26]. Based on the deep neural network method of multi-source data fusion, four sub-neural networks are constructed to extract the corresponding features of genes and diseases, to achieve pathogenic gene prediction [27]. He et al. [28] proposed an algorithm based on network enhancement to identify pathogenic genes. Different kinds of biological entities could provide complementary information for disease-causing genes prediction, hence it is essential to construct a heterogeneous networks using multi-omics data and represent nodes effectively for the prediction of pathogenic genes. However, it remains a challenge to integrate multiple biological entities to construct heterogeneous networks, effectively deal with the sparsity of biological networks, tap the complex cross-regulatory relationships among organisms, and improve the ability of disease gene prediction.

With the rapid development of artificial intelligence technology, various network representation learning methods have been proposed and applied to disease gene prediction. Most of the cutting-edge network representation methods, such as Node2vec and LINE, use biased random walk technology to obtain the similarity of nodes, which can effectively get the local and global features of the network. These network representation algorithms have achieved good performance in various scenarios [29, 30]. In recent years, attention mechanism has been widely used in Natural Language Processing (NLP) [31] and Computer Vision (CV) [32] to improve data correlation, enhance features and improve model accuracy. As well as attention has been successfully applied to bioinformatics. Such as Yu et al. [33] used single-head attention with a graph convolution network to predict drug targets. Snderby et al. [34] applied single-head attention to protein subcellular location prediction analysis. Because the single-head attention uses a single attention weight vector to weight the hidden state, the feature can only be mapped into a single space. It has some defects in interpreting the prediction results, and the performance is not very good. The multi-head attention composed of fully connected neurons is efficient and accurate in a calculation, and it presents powerful advantages in the most advanced NLP architecture, such as Transformer [35] and Bert model [36]. Wang et al. [37] also achieved the prediction of mRNA subcellular location by utilizing multi-head attention.

**Fig. 1:**
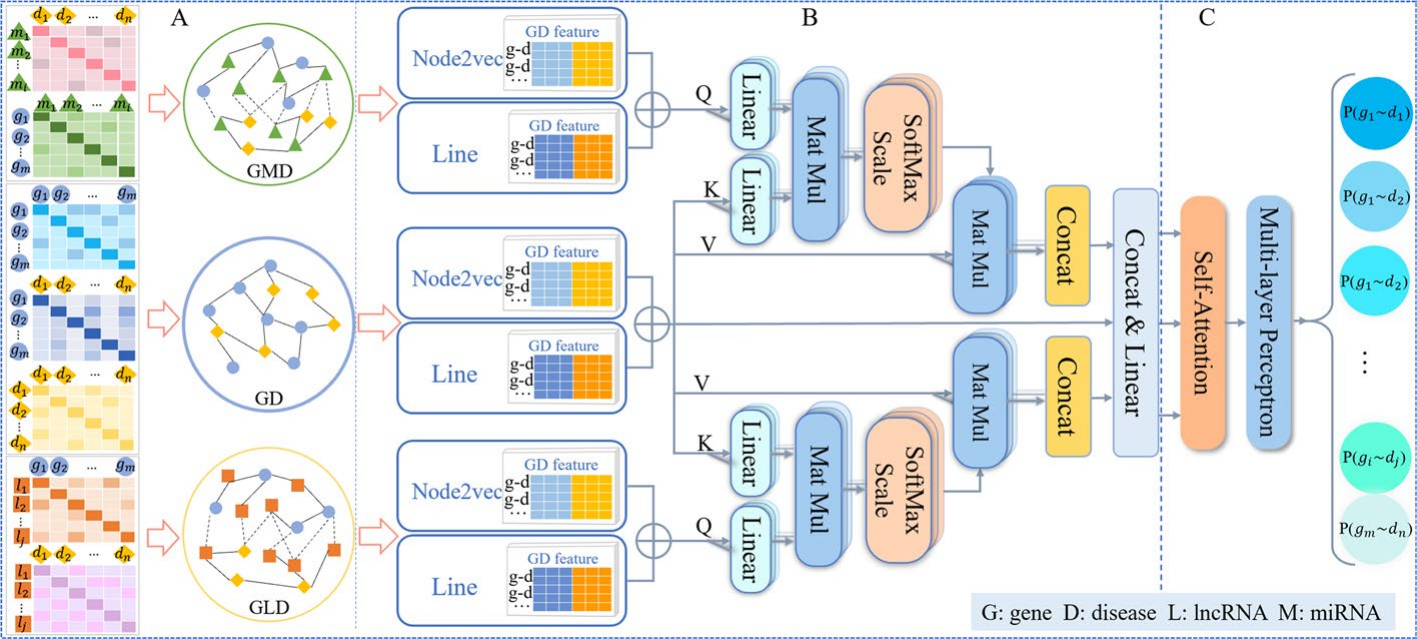
MHAGP framework. **A** Three heterogeneous networks are constructed based on the four integrated data sources (gene, disease, lncRNA and miRNA) and seven kinds of association (disease-miRNA, gene-miRNA, gene functional similarity, gene-disease, semantic similarity of disease, gene-lncRNA, disease-lncRNA). **B** The Node2vec and LINE algorithms are used to mine the biological association features of genes and diseases from three heterogeneous networks. The features extracted from the GMD and GLD networks are used to fusion the gene-disease association features in GD networks by multi-head attention. **C** Self-attention is introduced to predict the pathogenic gene in the multi-layer perceptron and output the gene-disease association score

Therefore, inspired by network representation learning algorithm and multi-head attention, to make more effective use of the complex regulatory relationship between multi-omics data, we propose a method called MHAGP for pathogenic gene prediction based on multi-head attention fusion. The overall model is shown in Fig. 1. Firstly, the MHAGP constructs three heterogeneous networks by integrating information from four biological entities, including gene, disease, lncRNA and miRNA, along with seven kinds of association, including disease-miRNA, gene-miRNA, gene functional similarity, gene-disease, semantic similarity of disease, gene-lncRNA, and disease-lncRNA. Then, Node2vec and LINE algorithms are used to mine the biological association features of gene and disease from three heterogeneous networks. The three features are fused by multi-head attention to enhance gene-disease association features. Finally, self-attention is introduced to predict the pathogenic gene in the multi-layer perceptron and output the gene-disease association scores. Through the evaluation of model performance, MHAGP is proved to be an effective method to merge the features of gene-disease association. The empirical results of five-fold cross-validation demonstrate that MHAGP outperforms all baselines. Besides, the assessment results of Alzheimer’s disease, lung cancer and myocardial infarction case studies verify the effectiveness and advantages of the proposed method.

The rest of the paper is organized as follows. Section II describes the implementation and architecture details of MHAGP. Section III introduces the datasets and analyzes the performance of MHAGP, compares it with eleven other competing algorithms, and makes a case study and some conclusions in section IV.

## Methods

Our model consists of three steps: (1) Network construction. We integrated four data sources and built three heterogeneous networks based on the complex regulatory relationship between biological characteristics. (2) Feature fusion. We use Node2vec and LINE algorithm to mine the original biological association features of genes and diseases from three heterogeneous networks and fuse the three gene-disease association features through multi-head attention. (3) Pathogenic gene prediction. Self-attention is introduced in the multi-layer perceptron to predict the pathogenic gene and output the gene-disease association score. The workflow is shown in Fig. 1.

### Construction of heterogeneous networks

We used four types of nodes and their seven associations to construct three heterogeneous biological networks, including GD ( gene-disease ), GMD ( gene-miRNA-disease ), and GLD ( gene-lncRNA-disease ) (see Fig. 1A). GD is constructed by integrating gene functional similarity, semantic similarity of disease and gene-disease association. Likewise, GMD is constructed by integrating gene-miRNA association and disease-miRNA association, and GLD is constructed by integrating gene-lncRNA association and disease-lncRNA association. If the association weight between biological nodes is greater than 0, an edge will be added. The constructed biological heterogeneous networks are undirected graphs.

### Extracting node features from networks

Graph representation learning is also called network representation. Its generation solves a series of difficulties in traditional manual feature extraction. In network modeling, it is an essential step in mapping node information to real vectors and can automatically learn the potential representation features of nodes. Node2vec [29] and LINE [30] are two avant-garde graphical representation algorithms. As an extension of the DeepWalk algorithm, Node2vec improves the sampling strategy of vertices in the Random Walk algorithm. It controls the random walk strategy by introducing two hyperparameters *p* and *q*. LINE algorithm optimizes the calculation method of similarity between nodes and considers the first-order and second-order similarity of nodes in the network graph. It can be applied to various types of networks (directed, undirected, weighted, and unweighted) and is suitable for large-scale networks.

In this study, we use Node2vec and LINE algorithms to extract the original feature representation of genes and diseases in three heterogeneous networks. For each node in the network, Node2vec and LINE get an e-dimensional real vector about genes and disease nodes according to the neighborhood information of the node. They finally get three different gene-disease association features of the two algorithms. Specifically, Node2vec and LINE obtain three gene-disease association feature matrices ($GD_{gd}, GD_{gmd} \text{ and } GD_{gld}$) from GD, GMD and GLD networks respectively. $GD_{gd} \in {\mathbb {R}}^{n \times 2e}$ is obtained by combining $G_{gd}^{i}=\left[ g^{1}, g^{2}, \cdots , g^{e}\right] \text{ and } D_{g d}^{j}=\left[ d^{1}, d^{2}, \cdots , d^{e}\right]$ vectors. $GD_{gmd} \in {\mathbb {R}}^{n \times 2e}$ is obtained by combining $G_{gmd}^{i}=\left[ g^{1}, g^{2}, \cdots , g^{e}\right] \text { and } D_{gmd}^{j}=\left[ d^{1}, d^{2}, \cdots , d^{e}\right]$ vectors. $GD_{gld} \in {\mathbb {R}}^{n \times 2e}$ is obtained by combining $G_{gld}^{i}=\left[ g^{1}, g^{2}, \cdots , g^{e}\right] \text{ and } D_{gld}^{j}=\left[ d^{1}, d^{2}, \cdots , d^{e}\right]$ vectors. Where *e* is the embedding dimension, and *n* is the number of gene-disease pairs.

The above feature representation is obtained simultaneously by Node2vec and LINE algorithms. Therefore, the feature matrices obtained by the two algorithms from three heterogeneous networks are fused separately to get: $GD_{g d}^{\prime } \in {\mathbb {R}}^{n \times 4e }, GD_{gmd}^{\prime } \in {\mathbb {R}}^{n \times 4e }, GD_{gld}^{\prime } \in {\mathbb {R}}^{n \times 4e }$.

### Multi-head attention fusion

Vaswani et al. [35] proposed a multi-head attention on the basis of attention. The purpose of the attention mechanism is to focus on the information that is more critical to the current task among the numerous input information, reduces the attention to other information, and even filters out irrelevant information, which can solve the problem of information overload and improve the efficiency and accuracy of task processing. The classic attention mechanism module consists of Query (Q), Key (K) and Value (V) operations. The core process is calculating the attention weight through *Q* and *K*, then acting on *V* to get the whole weights and outputs. Specifically, for the input matrices *Q, K* and *V*, the output vector is calculated as shown in Eq. (1).

1 $$\begin{aligned} Attention(Q, K, V)=Softmax\left( \frac{QK^{T}}{\sqrt{d_{k}}}\right) V \end{aligned}$$

Where $Q\in {\mathbb {R}}^{n \times d_{k}}, K \in {\mathbb {R}}^{m \times d_{k}}, V \in {\mathbb {R}}^{m \times d_{v}}$. Multi-head attention refers to multiple independent attention calculations, as an integration function, it integrates different knowledge generated from the same attention pooling. *Q, K* and *V* are transformed linearly, and each attention mechanism function is responsible for only one subspace in the final output sequence. That is, the so-called multi-head attention mechanism is a multi-group attention processing process of the original input sequence. Then the results of each group of attention are spliced together for a linear transformation to get the final output result. Given the query $Q \in {\mathbb {R}}^{d_{model} \times d_{\textrm{k}}}$, key $K \in {\mathbb {R}}^{d_{model} \times d_{k}}$ and value $V \in {\mathbb {R}}^{d_{model} \times d_{v}},\ d_{k}=d_{v}$, $W^{O} \in {\mathbb {R}}^{d_{model} \times hd_{v}}$, the multi-head is calculated by Eqs. (2)–(3).

2 $$\begin{aligned} head_{i}= & {} Attention\left( QW_{i}^{Q}, KW_{i}^{K}, VW_{i}^{V}\right) \end{aligned}$$

3 $$\begin{aligned} MultiHead(Q, K, V)= Concat\,\left( head_{1}, \ldots , head_{h}\right) W^{O} \end{aligned}$$

To better fuse the three different perspectives of gene-disease features extracted in the previous section, we use $GD_{gmd}^{\prime }$ and $GD_{gld}^{\prime }$ as auxiliary features of $GD_{gd}^{\prime }$ to fuse the data of gene-disease association features. The specific implementation details are shown in Fig. 1B. We get $GD_{gd_{-}m}^{att}$ through Eq. (4), as well as, obtains $G D_{g d_{-} l}^{att}$ through Eq. (5). *h* is set to 8 as suggested by [35]. To keep the original features of genes and diseases undistorted, we fuse $GD_{gd_{-}m}^{att}$, $G D_{gd_{-} l}^{a t t}$ and $G D_{gd}^{'}$ to obtain an enhanced gene-disease association feature matrix through Eq. (6), and recalculate the features again using self-attention in the next section.

4 $$\begin{aligned} GD_{gd_{-} m}^{a t t}= & {} MultiHead\left( G D_{gmd}^{\prime }, G D_{g d}^{\prime }, G D_{g d}^{\prime }\right) \end{aligned}$$

5 $$\begin{aligned} G D_{g d_{-}l}^{a t t}= & {} MultiHead\left( GD_{gld}^{\prime }, GD_{gd}^{\prime }, GD_{gd}^{\prime }\right) \end{aligned}$$

6 $$\begin{aligned} GD^{att}= & {} linear\left( concat\left( GD_{gd_{-} m}^{att},GD_{gd_{-}l}^{att},GD_{gd}^{\prime }\right) \right) \end{aligned}$$

### Gene-disease association prediction

We use the multi-layer perceptron as the last module of the model (see Fig. 1C). To effectively prevent the gradient disappearance problem in the model’s training, we use self-attention again to recalculate the feature values of all the available information. The specific implementation is as follows. Let $G D_{i}^{a t t}=\left[ g d_{i}^{1 }, g d_{i}^{2 }, \cdots , g d_{i}^{h}\right]$ represents the feature vector of the $\textit{i}$ th item in the gene-disease association feature after multi-head attention feature fusion enhancement, where $g d_{i}^{j} \in R, \forall j=1,2, \cdots , h$. By introducing attention parameter $H^{a t t} \in {\mathbb {R}}^{h \times h}, W^{att} \in {\mathbb {R}}^{h \times h}$ and bias parameter $b^{a t t} \in {\mathbb {R}}^{h \times h}$, calculate the attention score of each element in $GD_{i}^{att}$, as in Eq. (7).

7 $$\begin{aligned}&\alpha _{i}^{att}=softmax\left( H^{att} \cdot tanh \left( W^{att} GD_{i}^{att}+b^{a t t}\right) \right. \end{aligned}$$

Next, as shown in Eq. (8), the enhanced attention feature value is recalculated.

8 $$\begin{aligned}&GD_{i}^{att^{\prime }}=\alpha _{i}^{att} \otimes GD_{i}^{att} \end{aligned}$$

Where $\otimes$ represents pairwise multiplication.

The feature matrix $G D^{a t t^{\prime }}=\left[ G D_{i}^{a t t^{\prime }}\right]$ is used as the input $h^{\prime }$ of the perceptron module to score the relationship between genes and diseases. The number of nodes in the hidden layer is kept as the value of the hyperparameter $h^{\prime }$. The output layer sets a node and uses the sigmoid function to calculate the correlation score. The loss rate is measured to reduce over-fitting by calculating the binary cross entropy function. The cross entropy loss set as $L(Y), Y=\left[ y_1 ,\ y_2 , \cdots , y_n \right]$ is calculated as in Eq. (9).

9 $$\begin{aligned} L(Y)&=\frac{-1}{n} \sum \limits _{y_{i} \in Y} y_{i} log \left( p\left( y_{i}\right) \right) +\left( 1-y_{i}\right) log \left( p\left( 1-y_{i}\right) \right) \end{aligned}$$

The whole workflow of multi-layer perceptron in the prediction layer is summarized as in Eq. (10).

10 $$\begin{aligned}&y=Sigmoid\left( Linear \left( Relu \left( Linear \left( G D^{a t t^{\prime }}\right) \right) \right) \right) \end{aligned}$$

### Hyperparameters

**Fig. 2:**
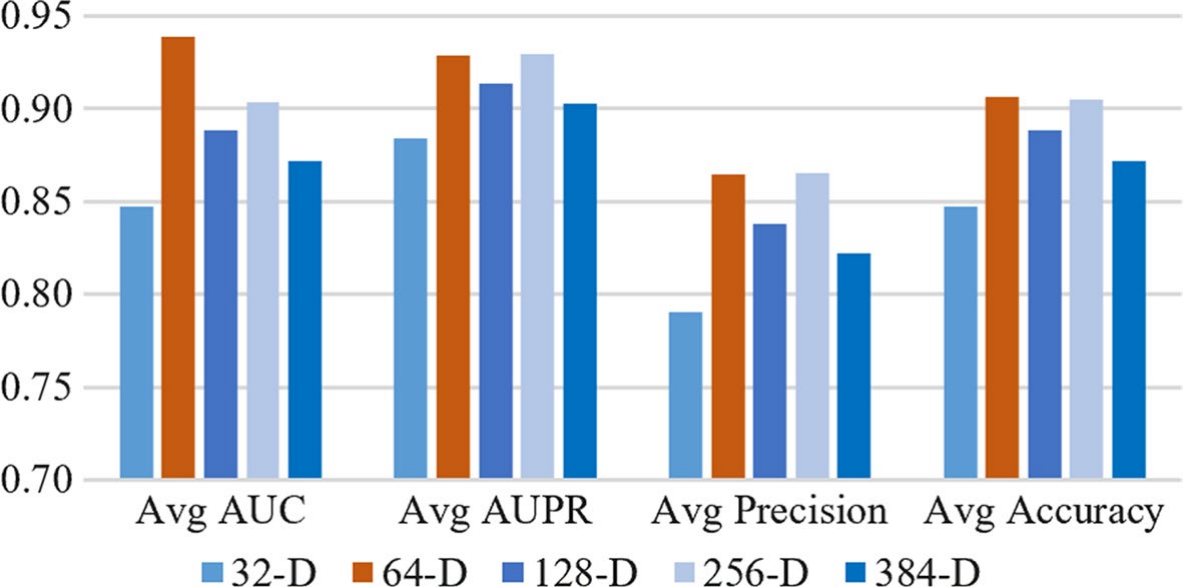
Dimension *e*-value comparison result

Different hyperparameters determine the robustness of the method in different modules. In this paper, referring to the parameter method set by [29], a loss rate of 0.2 is added among the hidden layers of the model, and the grid search method is used to adjust the hyperparameters. The dimension *e* embedded in Node2vec and LINE is selected from 32, 64, 128, 256. Other parameters in the network remain at default values. The data dimension remains unchanged when multi-head attention fuses the features of gene-disease association. The evaluation results are shown in Fig. 2. Our method performs best when $\textit{drop}$=0.2, $\textit{e}$=64, $\textit{lr}$=0.01, and $\textit{h}$=128. The results show that the model performance is poor if the $\textit{e}$ value is small. When *e* value is large, it will not affect the excellence of the model, but will reduce the training speed of the model. We adopt five-fold cross-validation to validate 10 epochs, 20 epochs, 30 epochs and 50 epochs, respectively, during model training. The model excellence tends to be stable after 30 epochs. Therefore, the model parameters in this paper is set as $batch_{-}size$=30, *epochs*=30.

## Results and discussion

In this section, at first, we have described the datasets and the evaluation metrics used in the model. Second, we have compared the performance impact of different data fusions on the model. Third, we have performed ablation experiments to assess the model’s accuracy. Fourth, we have selected twelve state-of-the-art methods as our baseline methods for comparison. Finally, we have performed candidate gene predictions for three diseases and have analyzed the results from the biological literature database and clinical perspectives.

### Experimental data sources

We use some datasets from Wang et al. [38]. The details are shown in Table 1. The gene-disease association mainly is from DisGeNET [39] and DISEASES [40]. The gene-lncRNA and disease-lncRNA association mainly come from the LncRNADisease2.0 [41], LncRNA2Target v2.0 [42], EVLncRNAs [43] and Lnc2Cancer 3.0 [44]. The gene-miRNA and disease-miRNA association come from the MNDR v3.0 [45] and MiRTarBase [46]. Through data error correction and data cleaning ( mainly including deleting duplicate, error and empty data ) on the data obtained from the database, then a unique ID is retained for each biomolecule. We get 7986 genes, 217 diseases, 814 lncRNAs and 2476 miRNAs.

**Table 1:** Experimental data sources

Name	Pair	Source	URL
Gene–Gene	56,310,502	Wang et al. [38]	–
Gene–Disease	37,277	DisGeNET [39]	https://www.disgenet.org
		DISEASES [40]	http://diseases.jensenlab.org
Gene-lncRNA	14,987	LncRNA2Target [42]	http://www.bio-bigdata.net/lnc2cancer
Gene-miRNA	216,934	MiR-TarBase [46]	https://mirtarbase.cuhk.edu.cn
Disease-Disease	43,273	Wang et al. [38]	–
Disease-lncRNA	3434	LncRNADisease 2.0 [41]	http://www.bio-bigdata.net/lnc2cancer
		EVLncRNAs [43]	https://www.sdklab-biophysics-dzu.net/EVLncRNAs2
Disease-miRNA	27,174	Lnc2Cancer 3.0 [44]	http://bio-bigdata.hrbmu.edu.cn/lnc2cancer
		MNDR v3.0 [45]	http://www.rnadisease.org

### Performance evaluation metrics

We use five-fold cross-validation to evaluate the performance of MHAGP and existing methods in gene-disease association prediction. In the experiment of MHAGP model, 80% of the subsets are used as training samples, and the remaining 20% are used as test samples. Gene-disease association prediction scores are generated upon test completion, and we rank them according to the prediction scores. According to the set threshold, when the prediction score is greater than the threshold, the corresponding prediction result is regarded as false positive (FP) or true positive (TP). Otherwise, it is viewed as a true negative (TN) or a false negative (FN). Specifically, the following evaluation indicators are used: True Positive Rate (TPR), False Positive Rate (FPR), Accuracy, Recall, Precision, F1-score and Area under Precision-Recall curve (AUPR). Receiver Operating Characteristic (ROC) uses TPR and FPR to draw the ROC curve under each value, and the area under the ROC curve is called the area under the ROC curve (AUC). The above calculation formula is shown in Eqs. (11)–(16).

11 $$\begin{aligned} TPR= & {} \frac{TP}{TP+FN} \end{aligned}$$

12 $$\begin{aligned} FPR= & {} \frac{TP}{FP+TN} \end{aligned}$$

13 $$\begin{aligned} Accuracy= & {} \frac{TP+TN}{TP+TN+FP+FN} \end{aligned}$$

14 $$\begin{aligned} Recall= & {} \frac{TP}{TP+FN} \end{aligned}$$

15 $$\begin{aligned} Precision= & {} \frac{TP}{TP+FP} \end{aligned}$$

16 $$\begin{aligned} F_{1-score}= & {} \frac{2}{\frac{1}{recall}+\frac{1}{\text {precision}}}=2 \times \frac{\text {recall} \times \text {precision}}{\text {recall}+\text {precision}} \end{aligned}$$

**Fig. 3:**
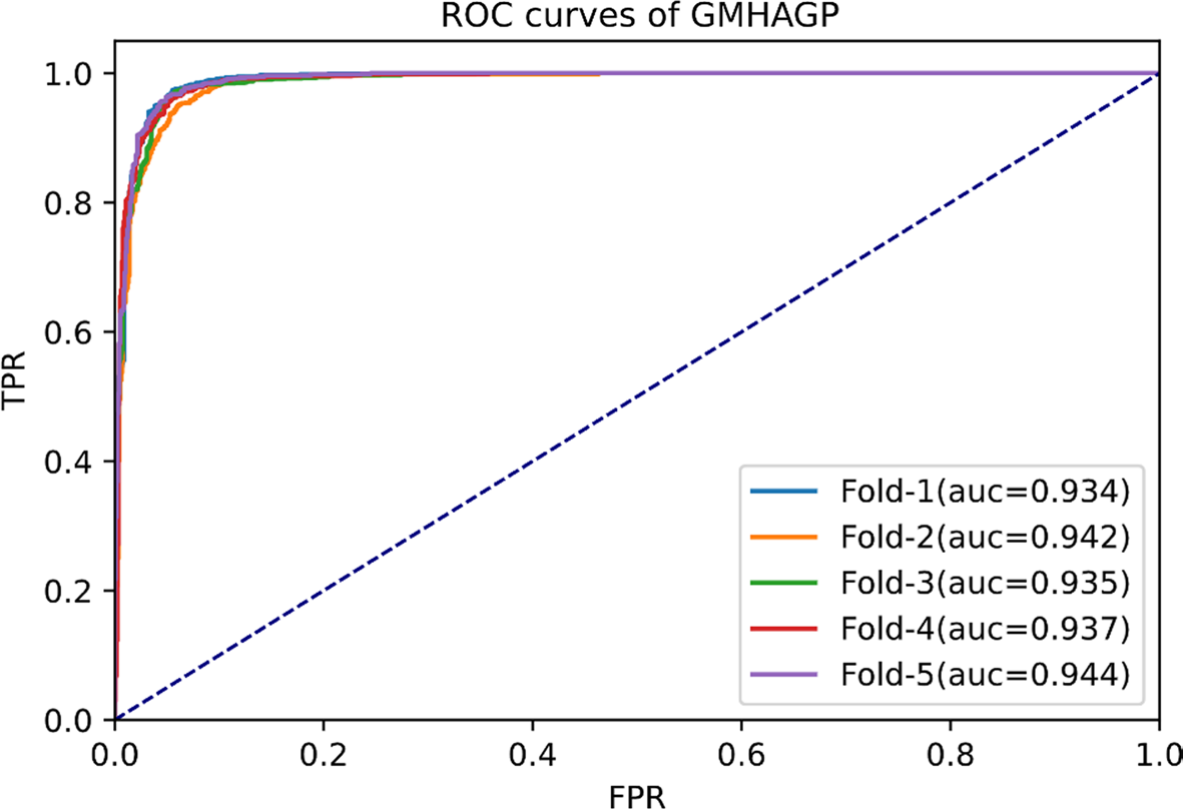
ROC curve for different value of five-fold cross-validation

According to the above formula, we draw the ROC curve (see Fig. 3) and evaluate the performance of MHAGP with the AUC value. The ROC curve changes over time. All known gene-disease associations were considered as positive samples in five-fold cross-validation. Conversely, unknown gene-disease association was considered negative sample. Since the number of positive samples in the data set is far less than that of negative samples, we use random sampling to repeat the experiment. According to the number of positive samples, we randomly sample an equal number of negative samples and report the average results with standard deviation. MHAGP has the best performance when the parameters are set to $e=64$, $h=128$, $h^{\prime }=384$, $lr=0.01$.

### Comparison of results of heterogeneous data sources

To compare the contribution of four biological data sources to the prediction accuracy of pathogenic genes, we use data sources under different combinations to compare the experimental results. The results are shown in Table 2. Bold values in the tables indicate the best performance. By using the association between gene, miRNA and disease, as well as the association between gene, lncRNA and disease to fuse the association between gene and disease for disease-causing gene prediction, the fusion of three heterogeneous network features can obtain more accurate results.

**Table 2:** Fusion results of different data sources

Data fusion	AUC (%)	Accuracy (%)	F1-score (%)	Precision (%)	AUPRC (%)
GD	84.44	84.44	83.50	77.99	88.01
$\hbox {GD}+\hbox {GMD}$	88.15	88.15	86.11	83.37	91.00
$\hbox {GD}+\hbox {GLD}$	87.50	87.49	87.34	81.02	90.06
$\hbox {GD}+\hbox {GMD}+\hbox {GLD}$	**93**.**84**	**90**.**64**	**90**.**84**	**86**.**48**	**92**.**86**

### Ablation study

**Fig. 4:**
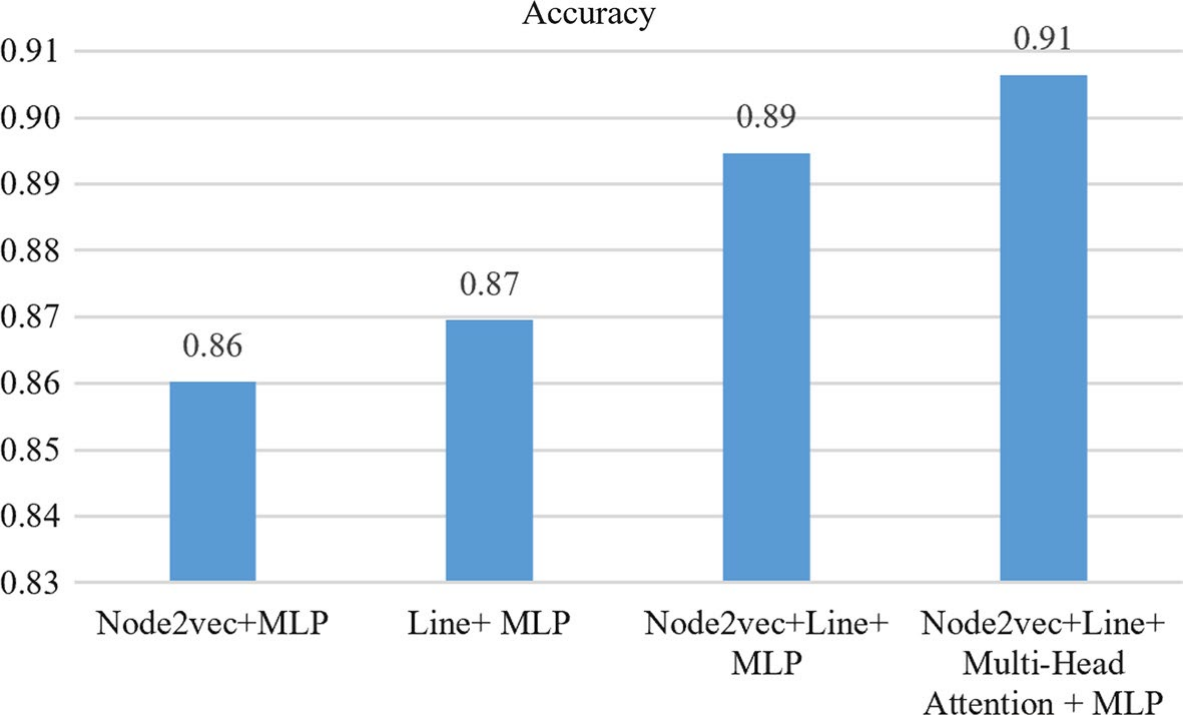
Accuracy of the model based on feature combinations

To analyze the influence of the feature representation learned by MHAGP on the prediction model’s performance, we have made experimental comparisons on the combination of different modules. Figure 4 shows the results of four ablation experiments. The average accuracy given by the MHAGP model is 0.91 (± 0.0002), and the overall index is the highest among the four combinations. The results show that the accuracy of the prediction model is significantly improved by introducing multi-head attention to feature enhancement.

### Comparison with other methods

To evaluate the feasibility of MHAGP, we compare our model with the seven excellent ML methods proposed by [21], two cutting-edge graph neural network models [47, 48], and three disease-causing gene prediction methods proposed in recent years [25, 49]. The results of the model performance comparison are shown in Table 3. The results of our model are best in all six-evaluation metrics among seven machine learning methods, including Logistic Regression (LR), Random Forest (RF), support vector machines (SVM), Decision tree, KNN, Gradient Boosting (GB) and Multi-layer Perceptron (MLP). Among the two graph neural network models, the Graph Attention Networks (GAT) [47] model is based on Graph Convolutional Networks (GCN). Heterogeneous Graph Attention Network (HAN) [48] turns a heterogeneous network with different meta-paths into a homogeneous network with different edge weights and then uses the HAN model to predict the association between nodes. Compared with three state-of-the-art pathogenic gene prediction models, PINDeL [25] based on graph convolutional neural network, dgMDL [49] based on DBN and network enhancement-based DGHNE [28], MHAGP shows better performance among the six indicators. Therefore, the model in this paper shows the best performance among all baseline methods, as shown in Table 3.

**Table 3:** The overall performance of compared to the existing methods

Method	AUC (%)	Accuracy (%)	F1-score (%)	Precision (%)	Recall (%)	AUPRC (%)
LR	82.21	82.11	79.62	76.08	83.51	86.49
RF	84.38	84.39	81.79	78.89	84.91	88.24
SVM	82.98	82.98	80.62	77.04	84.56	87.13
DT	70.35	78.25	66.39	64.48	68.42	77.69
KNN	79.67	79.67	78.64	72.78	85.54	84.75
GB	85.09	85.09	82.80	79.56	86.32	88.70
MLP	83.51	83.51	80.16	78.31	82.11	87.77
GAT	82.35	91.42	85.36	82.03	88.97	82.00
HAN	90.57	**94**.**75**	85.68	82.6	88.99	89.79
PINDeL	81.42	83.45	81.84	79.67	84.13	86.05
dgMDL	87.82	87.82	85.46	83.22	87.82	90.87
DGHNE	78.94	78.94	66.79	78.94	57.89	89.47
MHAGP	**93**.**84**	90.64	**90**.**84**	**86**.**48**	**95**.**66**	**92**.**86**

### Case studies

To further evaluate MHAGP, we rank gene-disease pairs based on the relevant probabilities calculated by the model. We predict and analyze three specific diseases (Alzheimer’s disease, lung cancer and myocardial disease) genes. Firstly, we train the MHAGP model using a data set containing all gene-disease associations except the associations between three diseases and genes. Secondly, we use the trained model to predict the association probability of three diseases with candidate genes and rank them, respectively. Finally, the top 20 candidate genes of the three disease prediction results were analyzed and demonstrated through scientific publications and the latest updated data of online biological databases such as OMIM and DisGeNET, as shown in Table 4. The evidence column indicates the associated citations from some reference databases and literature.

**Table 4:** Top 20 MHAGP predicted genes associated with three diseases

Rank	Alzheimer disease	Evidence	lung cancer gene	Evidence	Myocardial disease	Evidence
1	HLA-B	PMID: 17176470; DisGeNET	CCL2	PMID: 33253790; DisGeNET	COTL1	PMID: 32730836; DisGeNET
2	RPLP0	PMID: 35615586; DisGeNET	CXCL1	PMID: 31998654; DisGeNET	CDKN1A	PMID: 31919418
3	TGFB1	PMID: 31792364; DisGeNET	FGFR1OP	PMID: 26905588; DISEASES	GSPT1	DISEASES
4	MEST	PMID: 34625606	TNF	PMID: 35016421;	EIF2B4	CREEDS
5	ANXA4	*	FAM189A2	OMIM	PCBP1	PMID: 26116532
6	ITGB2	PMID: 30787942; DisGeNET	IL6	PMID: 32020709; DisGeNET	PTGES2	DisGeNET
7	CDKN2A	PMID: 34219731; OMIM	RAB7A	PMID:35449308; CREEDS	COL18A1	OMIM
8	ATM	PMID: 27022623; OMIM	IL1B	PMID: 23784458; DisGeNET	SERINC5	DisGeNET; DISEASES
9	ACTB	PMID: 24628925	CDH13	PMID: 29416663; DisGeNET	SRSF2	PMID: 34298011
10	PYCARD	PMID: 33273068	TTC19	CREEDS	CCL5	PMID: 28987763; DisGeNET
11	ACTA2	PMID: 34916831	CXCL5	PMID: 29200871; DisGeNET	TUBB6	DisGeNET
12	MYC	PMID: 33729395	PARP1	PMID: 33284833; DisGeNET	PLAC8	CREEDS
13	TMBIM1	DISEASES	LRP11	CREEDS	PMPCB	DISEASES
14	SPARC	PMID: 33400467; DISEASES	CCL11	PMID: 33452453; DisGeNET	MBNL1	PMID: 33295096; DisGeNET
15	ALDH2	PMID: 27808372; DisGeNET	COL3A1	PMID: 32300359; DisGeNET	PSMA4	PMID: 35952493
16	TP53	PMID: 29842899; DisGeNET	CCL7	PMID: 30214518; DisGeNET	PTGS2	PMID: 35311466; PTGS2
17	PTEN	PMCID: PMC7654589	ZEB1	PubMed: 31719531;OMIM	PTMA	PMID: 33398012
18	RPL10	DisGeNET; OMIM	ESR1	PMID: 35281414; DisGeNET	AR	PMID: 26769913; DisGeNET
19	PDCD4	PMID: 32474742; DisGeNET	ARRDC4	CREEDS	CCNL1	*
20	RPL11	PMID: 33541173	BTN2A2	*	GPX3	PMID: 35073209

In the prediction results of Alzheimer’s disease, 18 genes (90%) have been related to reference databases and literature evidence. Among the two newly predicted candidate genes, the latest research [50] shows that the RPL11 gene is significantly up-regulated in Alzheimer patients. As a tumor invasion-enhancing gene, the ANXA4 gene can promote trophoblast invasion in preeclampsia patients through PI3K/Akt/eNOS pathway [51]. In the prediction results of lung cancer, it is surprising that the reference database confirmed 19 genes (95%). Our predicted novel gene BTN2A2 is a T-cell immune regulatory molecule, which can be further studied as a potential gene related to lung cancer in the future. 17 (85%) candidate genes highly correlated with myocardial infarction predicted by MHAGP were confirmed by the reference database. Among the other three predicted new genes, the OMIM database showed that the COL18A1 gene was transcribed in multiple organs and was related to vascular endothelial inhibitors. For the AR gene, [52] showed that the lack of androgen would cause increased lipid accumulation and aggravate atherosclerosis, but AR could inhibit the progression of atherosclerosis. As a potential tumor gene, CCNL1 is not directly related to myocardial infarction, so that it can be further explored as a candidate gene for myocardial infarction.

Due to limited research on bio-molecules, the new genes of the three diseases predicted in this paper can be used as new suggestions for biological laboratory validation. Further research on their biological functions and regulatory mechanisms can provide better diagnosis and treatment schemes for clinical medicine. Through association prediction of three disease candidate genes, the performance of the MHAGP model in new association prediction is demonstrated. Our approach has potential value in discovering novel genes associated with complex human diseases.

## Conclusions

In this work, we propose a method to predict the pathogenic genes using multi-head attention fusion. Firstly, the heterogeneous biological information networks of disease genes are constructed by integrating multiple biomedical knowledge bases. Secondly, two graph representation learning algorithms are used to capture the feature vectors of gene-disease node pairs from the networks, and the gene-disease association feature pairs are fused by introducing multi-head attention. Finally, we use multi-layer perceptron model to predict the gene-disease association. The MHAGP model outperforms all other methods in comparative experiments. Case studies of Alzheimer, lung cancer and myocardial disease also show that MHAGP can predict genes potentially associated with the disease. In the future, more types of biological entity data, such as gene-drug, disease phenotype-gene ontology, etc., can be added to expand the amount of information in heterogeneous biological networks and achieve more accurate prediction. In addition, the MHAGP model can also be used for potential tasks such as gene-drug association prediction and drug-disease association prediction. Therefore, MHAGP has strong expansibility, which can help to study the mechanism of gene action in diseases in the future.

## Data Availability

The code and data used in this study are freely downloadable at https://github.com/Bio503/MHAGP.
